# Limitations of the glycaemic index and the need for nuance when determining carbohydrate quality

**DOI:** 10.1093/cvr/cvab312

**Published:** 2021-10-07

**Authors:** Mitch Kanter, Siddhartha Angadi, Julie Miller-Jones, Katherine A Beals

**Affiliations:** Alliance for Potato Research and Education, Chicago, IL, USA; Department of Kinesiology, University of Virginia, Charlottesville, VA, USA; Department of Nutrition, St. Catherine’s University, St. Paul, MN, USA; Nutrition & Integrative Physiology, University of Utah, Salt Lake City, UT, USA

In the recent review study by Riccardi *et al*., the study’s notable focus on the glycaemic index (GI) to inform broad dietary guidance is troubling given GI’s demonstrable limitations as a tool for predicting health risk. In 2019, Reynolds *et al*.^1^ observed the relationship between GI and clinical outcomes was consistently graded as low to very low. Furthermore, a recent analysis demonstrated the inconsistent link between GI and hard clinical endpoints across populations that likely have divergent risk profiles (e.g. for developed vs. developing countries).^2^^,^^3^

As Riccardi *et al*.^4^ previously noted in an earlier publication, intervention studies on the effects of GI on cardiovascular disease outcomes are not clear, and while GI may have some utility for people with type 2 diabetes, it has not shown to be particularly useful as a standalone metric. Indeed, multiple studies have demonstrated GI’s wide intra- and inter-individual variability, suggesting it cannot be considered a reliable nor generalizable measure of glycaemic impact on health outcomes.^5–9^ In the most comprehensive evaluation of post-prandial glycaemic responses with controlled meals to date, Zeevi *et al*.^10^ observed a roughly five-fold interpersonal variability in glycaemic response to bread between the bottom and top 10% of participants. This degree of inter-individual variability would disqualify the use of any other biomarker of health or disease status.

Lifestyle factors, many of which are difficult to control even in lab settings, can affect one’s glycaemic response to a food (e.g. prior exercise, stress, lack of sleep, composition of previous meal(s), etc.).^8^ There is also significant GI variability within food categories and across geographic locations as well.^11^^,^^12^ Variables such as food variety, growing conditions, small changes in meal preparation, and even degree of mastication can impact GI.^13–15^

Furthermore, GI tables are developed based on consumption of 50 g of available carbohydrate of a food. This quantity of carbohydrate is often difficult to attain in ad lib food settings. For example, when comparing rice to beets—both classified as high GI foods—the 50 g threshold for available carbohydrate is a reasonable measure for rice (there are 53 g of carbohydrate per cup and most of it is available). However, the GI for beets is 64, which has little real-world meaning as there are only 13 g of carbohydrate per cup of beets; to reach the 50 g threshold one would need to consume more than 4 cups of beets.^16^

On the basis of GI alone, the authors pointedly call out potatoes as a food to limit, yet evidence indicates that potatoes in many forms (despite its GI, which varies significantly depending on variety and cooking preparation), produce different (generally more beneficial) effects on food intake, satiety, blood glucose, and insulin responses than pasta, which has a lower GI.^17^

All of these issues speak to the impracticality of GI as a metric for carbohydrate quality in real-world settings. A recent review by the US Academy of Nutrition and Dietetics noted that the GI may be a useful tool in some contexts, but acknowledged it is ‘an imperfect system’, noting one of its major flaws is that it assesses glycaemic impact on an empty stomach and when consumed without any other foods or condiments.^16^

Lastly, the use of GI seems counter to the authors’ call for a ‘meal pattern’ vs. an individual nutrient approach, a perspective widely supported among health professionals and government agencies.^18^ Yet, GI is not a metric for assessing the healthfulness of meal patterns, but rather, analyzes carbohydrates in isolation of all other dietary factors. Recently published reviews and perspectives call into question its use as a marker of diet quality^19^ and strongly recommend departing from such reductionist assessment methods and have called for a more holistic approach to evaluating the quality of carbohydrate foods.^20^^,^^21^

**Conflict of interest:** M.K. serves a a consultant for the Alliance for Potato Research & Education (APRE). K.A.B is consultant for Potatoes USA. 
